# Index extraction for electromagnetic field evaluation of high power wireless charging system

**DOI:** 10.1371/journal.pone.0180019

**Published:** 2017-07-14

**Authors:** SangWook Park

**Affiliations:** EMI/EMC R&D Center, Reliability & Safety R&D Division, Korea Automotive Technology Institute, Cheonan, Korea; University of California San Francisco, UNITED STATES

## Abstract

This paper presents the precise dosimetry for highly resonant wireless power transfer (HR-WPT) system using an anatomically realistic human voxel model. The dosimetry for the HR-WPT system designed to operate at 13.56 MHz frequency, which one of the ISM band frequency band, is conducted in the various distances between the human model and the system, and in the condition of alignment and misalignment between transmitting and receiving circuits. The specific absorption rates in the human body are computed by the two-step approach; in the first step, the field generated by the HR-WPT system is calculated and in the second step the specific absorption rates are computed with the scattered field finite-difference time-domain method regarding the fields obtained in the first step as the incident fields. The safety compliance for non-uniform field exposure from the HR-WPT system is discussed with the international safety guidelines. Furthermore, the coupling factor concept is employed to relax the maximum allowable transmitting power. Coupling factors derived from the dosimetry results are presented. In this calculation, the external magnetic field from the HR-WPT system can be relaxed by approximately four times using coupling factor in the worst exposure scenario.

## Introduction

Recently, wireless power transfer (WPT) technique using electromagnetic resonance phenomena has been proposed by MIT research team [1]. This technique can achieve efficient WPT over relatively long distances between transmitter and receiver compared to conventional inductive power transfer techniques [2–8]. The highly resonant (HR) WPT systems typically operate in the low MHz range of approximately 1 to 20 MHz, because this range is proper to reduce wire loss caused by the skin depth and to realize high resonant coils. Since this HR-WPT technique has been reported, there has been increasing interest in the commercialization of HR-WPT system.

The HR-WPT systems using the strong reactive near fields may induce high internal electric fields in a human body in the vicinity to the systems. In particular, the HR-WPT systems can produce excessively higher fields than those used in wireless communications systems. Therefore, it is important to evaluate the internal electric fields induced in the human body in the vicinity of the HR-WPT system and to confirm compliance with international safety guidelines. The related studies have been conducted in [9–11].

For commercialization, one of the most interesting factors is the maximum allowable transmitting power under the compliance with the safety guidelines. Safety guidelines/standards for non-ionizing electromagnetic field exposure of occupational and general public have been provided by the International Commission on Non-Ionizing Radiation Protection (ICNIRP 1998 [12] and 2010 [13]) and the Institute of Electrical and Electronics Engineers (IEEE C95.1–2005 [14]). Test methods and procedures for electromagnetic exposure safety of products have been provided by IEEE ICES/Technical Committee (TC) 34 and International Electrotechnical Commission (IEC) TC 106. There is no experimental compliance procedure for electromagnetic fields exposure between 400 kHz and 30 MHz except for IEC 62311 [15], which describes only general aspects. Thus, when evaluating the compliance of non-uniform electromagnetic field exposure with the safety guidelines/standards, the measured maximum external field strength is first compared to a limit of reference level or maximum permissible exposure, provided by ICNIRP guidelines and IEEE standards. Generally, if the measured external fields are not satisfied for the reference levels, the internal fields induced in the human body should be obtained and compared to the limit of the basic restrictions provided by the safety guidelines/standards. Above 100 kHz, these guidelines/standards provide basic restrictions for electromagnetic fields in metric of the specific absorption rate (SAR) in the body to prevent whole-body heat stress and excessive localized tissue heating. For the general public, a whole-body averaged SAR limit of 0.08 W/kg and a localized SAR limit of 2 W/kg averaged over 10 g of tissue are recommended. The frequency range of interest for HR-WPT system is in a transition region where the other basic restriction is provided to protect electrostimulation of nerve tissues below 10 MHz. It is difficult to comply with the reference level in the case of applications requiring high transmitting power such as electric vehicle charging. Therefore, the use of coupling factor concept, which is defined by IEC 62311, will be the reasonable procedure to evaluate the compliance by measuring external field and comparing to the reference levels.

In this paper, the HR-WPT system is designed to operate at 13.56 MHz frequency, which is one of Industry-Science-Medical band. The dosimetry for the system is conducted by the two-step approach [10] in the condition of misalignment as well as alignment between transmitter and receiver, and along the various location of human body with respect to the system. Coupling factors are derived from the dosimetry results for the HR-WPT system.

## Materials and methods

### HR-WPT system

**Fig 1:**
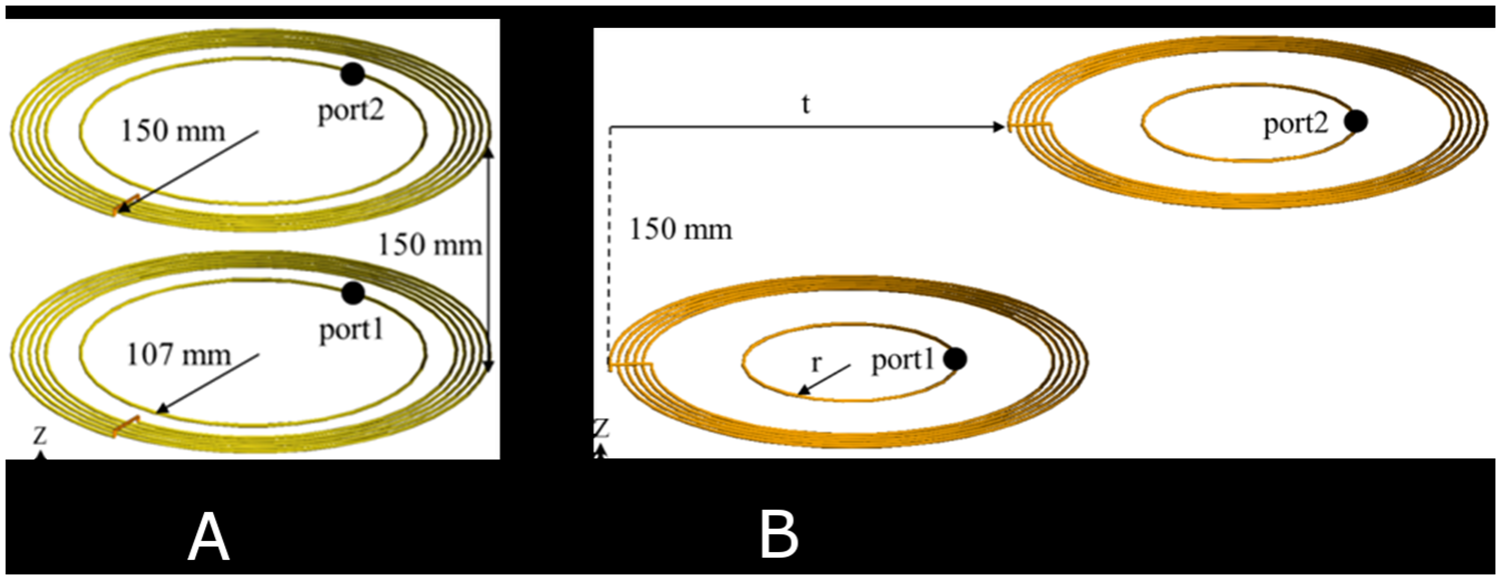
Configuration of highly resonant wireless power transfer system. (a) alignment, (b) misalignment.

A highly resonant wireless power transfer (HR-WPT) system is compactly designed to operate at 13.56 MHz frequency (Fig 1). The HR-WPT system consists of two resonant coils and two feeding loops. The inner loops play the role of a matching circuit. The outer coil radius of the HR-WPT system and the power transfer distance are set by 150 mm. Copper wire with a radius of 2 mm is used for the system. In the conditions of misalignment (Fig 1b) as well as alignment (Fig 1a) between transmitter and receiver, the power transfer efficiency is investigated because of often the situation when charging. The power transfer efficiency in the misalignment case generally decreases without matching. However, the practical HR-WPT system should be matched for the power transfer efficiency. In this work, the power transfer efficiency is investigated with various misaligned distances, t, in the matching and mismatching conditions. The designed HR-WPT system is simulated by commercial electromagnetic solver (FEKO) based on the hybrid technique of method of moment (MOM) and the finite element method (FEM), to obtain the power transfer efficiency and the electric and magnetic field nearby the system.

### Computational method

To investigate the compliance with the safety guidelines for the HR-WPT system used in the vicinity of the human body, dosimetry for the system is conducted with the anatomically realistic Japanese adult male model, TARO [16]. This human model possesses 2-mm spatial resolution and 51 tissues and organs, based on the accumulated magnetic resonant imaging (MRI) images of adult Japanese volunteer. The electrical properties of the TARO model are taken from Gabriel’s Cole-Cole models [17]. The SAR in the human body is calculated by using a two-step computation method [10]. In the first step, the electric fields, which are produced by the HR-WPT system in the absence of the human body, are calculated by using the HFSS. In the second step, by considering the fields obtained in the previous step as the incident fields, the SARs in the human body are computed by using the scattered-field FDTD method [18]. It should be noted that this two-step computation method does not include the interaction effects between the HR-WPT system and the human body. However, the interaction effects are negligible because of the weak backscattering from the human body [19].

### Coupling factor

The applications of WPT technique will gradually require higher transmitting powers. This means that a human will be exposed to strong fields in proximity to the HR-WPT system. This condition makes the compliance of the safety guidelines to be difficult. The reasonable procedure is necessary for both companies and consumers. Employing coupling factor concept is able to increase the maximum allowable transmitting power under the compliance of the safety guidelines. That is, the factor compensates the spatial-peak field strength measured at one point for equivalent comparison to the basic restrictions. The general definition of the coupling factor in [15] is employed for the compliance with a HR-WPT system. In this work, the external field strength to compare with the reference levels is considered only the magnetic field because the magnetic field strength is dominant rather than magnetic field strength in a WPT system, which the electromagnetic field energies is transferred by magnetic coupling. Coupling factors for the SAR and 2mm-spatial peak SAR have been derived by the following equations, respectively.

$$\kappa =\left(\frac{\sqrt{SAR_{cal}}}{H_{cal}}\right)/\left(\frac{\sqrt{SAR_{lim}}}{H_{lim}}\right)$$ (1)

$$\kappa_{peak}=\left(\frac{\sqrt{SAR_{cal\_peak}}}{H_{cal}}\right)/\left(\frac{\sqrt{SAR_{lim}}}{H_{lim}}\right)$$ (2)

where *SAR*_*cal*_ and *SAR*_*cal*_*peak*_ are the peak values of the SAR averaged over 10 g of tissue and the SAR averaging volume of 2mm× 2mm × 2mm cube in the human model, while *SAR*_*lim*_ is their basic restrictions provided by the safety guidelines. *H*_*cal*_ and *H*_*lim*_ are the spatial maximum magnetic field calculated by HFSS and the reference level for the magnetic field strength in the safety guidelines, respectively. *κ*_*peak*_ is always larger than κ because *SAR*_*cal*_*peak*_ averaging 2-mm spatial volume is larger than *SAR*_*cal*_ averaging 22-mm spatial volume at the peak point. Employing *κ*_*peak*_ expects the conservative estimation rather than κ. When measuring the magnetic field for a practical WPT system, the calculated value of *H*_*cal*_ can be replaced by measured value. The measure or calculated magnetic field strength multiplied by coupling factors is relaxed and can be directly compared to the reference level.

## Results and discussion

### Characteristics and analysis of the HR-WPT system

**Fig 2:**
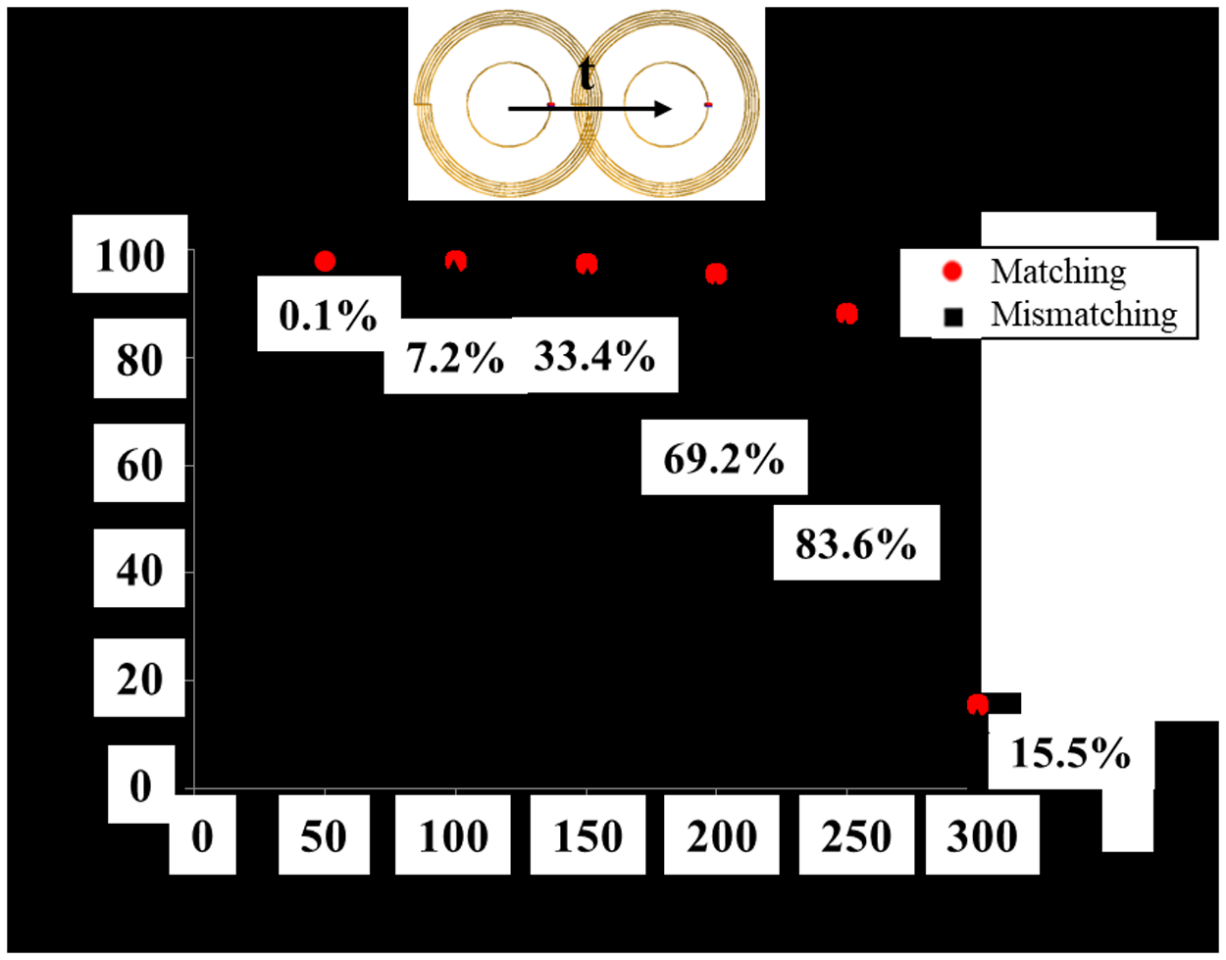
Power transfer efficiency comparison of HR-WPT system along with matching and mismatching condition.

The power transfer efficiency (|*S*_21_|^2^) of the HR-WPT system designed here is 98% in the alignment case. The power transfer efficiencies along with the misaligned distance (t) are shown in Fig 2, for matching and mismatching condition. The matching condition can be achieved by adjusting the radius of inner loop (r).

The input impedance is changed with the coupling coefficient between the inner loop and the outer coil. This coupling coefficient depends on the mutual magnetic flux between the outer coil and the inner loop. Thus, we can easily find the matching condition as the input impedance is swept by adjusting the loop size. Without the matching, the power transfer efficiency rapidly drops as the misaligned distance increases. However, the power transfer efficiencies improve with the matching compared to those without the matching. Specially, the efficiency improvement of 83.6% is the best at misaligned distance of 250 mm. At this misaligned distance, the difference of radiation from the HR-WPT system between the alignment and misalignment situation is the largest among the other misaligned distance.

**Fig 3:**
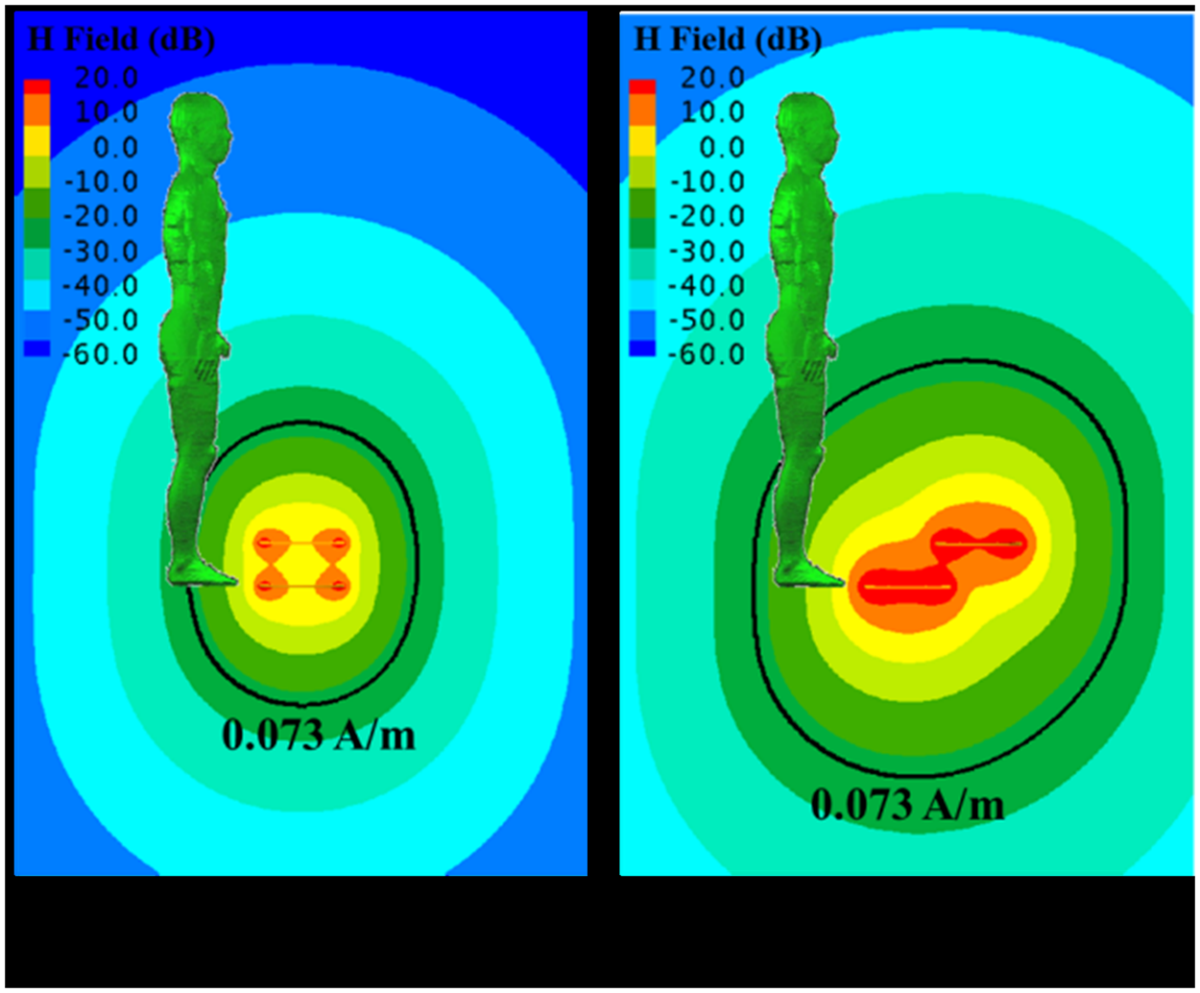
Magnetic field strength distribution nearby the high resonant wireless power transfer system. The black solid line indicates the reference level defined as ICNIRP guideline.

**Fig 4:**
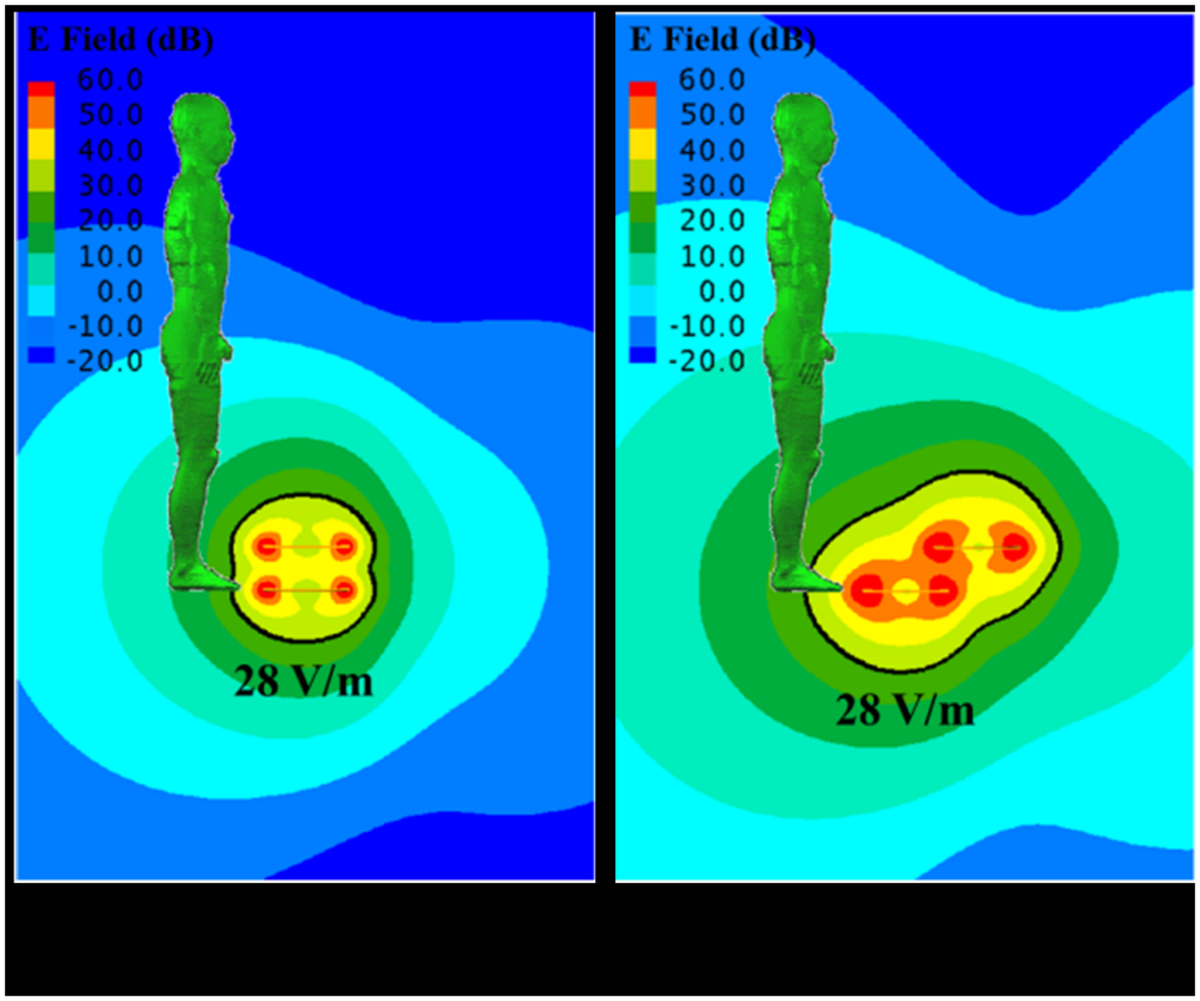
Electric field strength distribution nearby the high resonant wireless power transfer system. The black solid line indicates the reference level defined as ICNIRP guideline.

The electric field strength and magnetic field strength for reference level of the 1998 ICNIRP guideline are 28 V/m and 0.073 A/m, respectively, at 13.56 MHz frequency [12]. The electric and magnetic field strength nearby the HR-WPT system are calculated to investigate the compliance with the reference level of the system. The magnetic and electric field strength distributions nearby the system of 1 W input power for the alignment (case1) and misalignment (case2) are shown in Figs 3 and 4, respectively, with the reference level, which is indicated by black solid line. The field distributions are the results in the absence of human body. However, the field distribution results are shown with the human body model to confirm the compliance with the ICNIRP guidelines. As shown in the results, the area over the reference level of magnetic field strength is wider than that of electric field strength. We can also find that the area over the reference level in the misalignment condition is wider than in the alignment condition. This result suggests that we should consider dosimetry for the misalignment situation.

### Dosimetry and coupling factors

#### Alignment and misalignment

**Fig 5:**
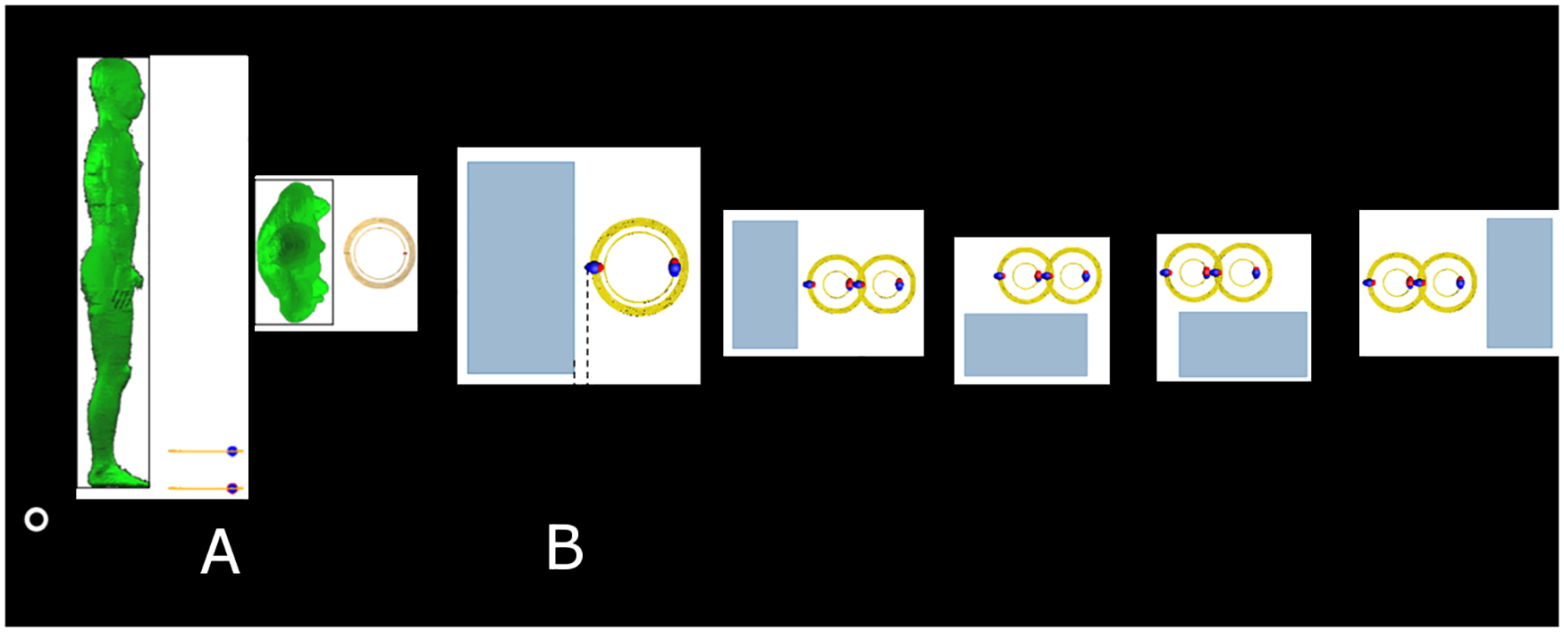
(a) configuration of human voxel model with respect to a highly resonant wireless power transfer system, (b) exposure scenarios for alignment and misalignment conditions.

WPT technology is being applied in various fields. In the case of an electronic device requiring a low output power, the level of electromagnetic field radiation is not so large. However, in high-power applications such as electric vehicles, the emission of electromagnetic fields is very serious. In this paper, the exposure in the proximity of the WPT systems on the ground is investigated, assuming the application of the most serious electric vehicle. Anatomically realistic human voxel model, TARO, stands in front of a HR-WPT system as shown in Fig 5(a). Dosimetry scenarios are one case for alignment and four cases for misalignment as shown in Fig 5(b). In order to investigate dosimetry for misalignment with regard to that for alignment, the human body is located nearby transmitter (case2 and case3) and nearby receiver (case4 and case5) because strong magnetic and electric fields are constructed around the transmitting and receiving resonant coils. At these cases, distance between a HR-WPT system and a human body is 50 mm. SARs results are shown in Table 1. These results show that the SAR in a misalignment case is generally larger than that in alignment case. It is found that the SAR in case 2 is the largest, that is to say the worst case among the exposure scenarios.

**Table 1:** Specific absorption rate in a human body for alignment and misalignment.

Cases	SAR_peak_ (mW/kg)	SAR_10g_ (μW/kg)	SAR_wb_ (μW/kg)
1	1.37	162	2.09
Tissue name	skin	fat	-
2	13.52	1573	16.87
Tissue name	skin	fat	-
3	1.29	199	6.32
Tissue name	skin	muscle	-
4	1.10	165	6.17
Tissue name	skin	skin	-
5	1.02	96.7	6.21
Tissue name	skin	fat	-

SAR_peak_ represents the peak value of the SAR averaging volume of 2mm × 2mm × 2mm

SAR_10g_ represents the peak value of the SAR averaged over 10 g of tissue

SAR_wb_ represents the whole-body SAR

**Fig 6:**
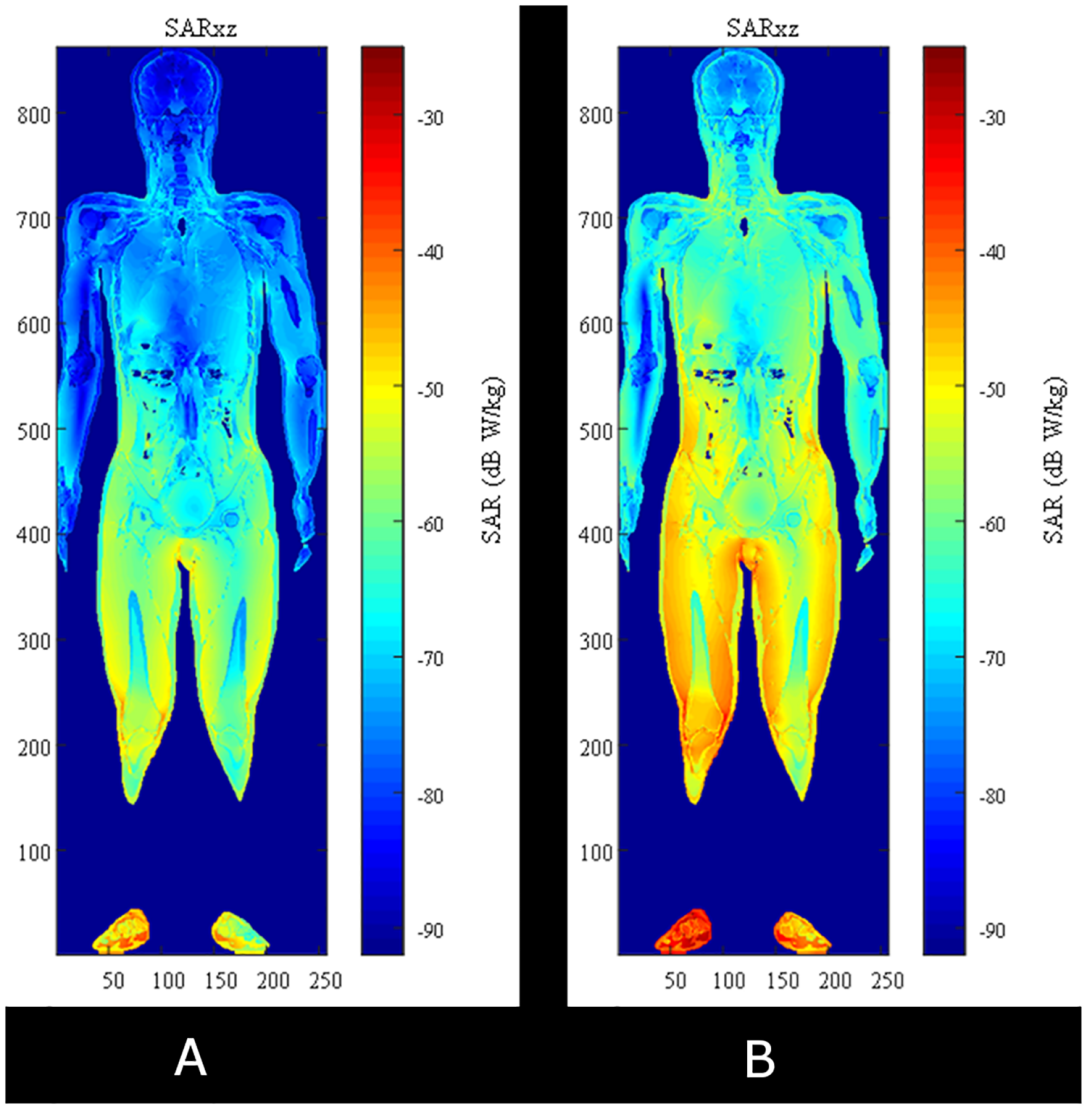
SAR distributions of cross section in xy plane. (a) case 1, (b) case 2.

**Fig 7:**
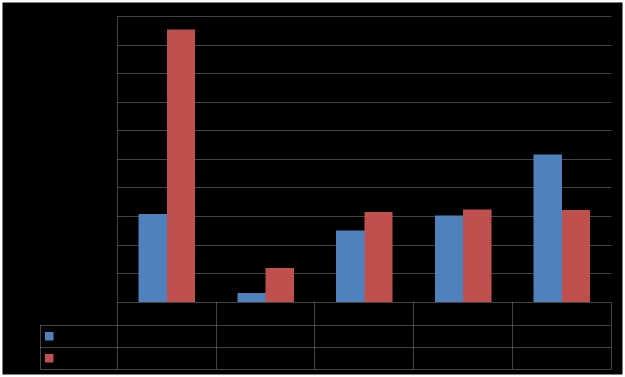
Maximum allowable power (MAP) for cases 1 to 5.

The log scale SAR distributions for case 1 and case 2 are shown in Fig 6, respectively. As you can see, the SARs are strongly distributed at lower half of body because a HR-WPT system is close to feet of human model. It is also found that the distributed SAR for case 2 is entirely stronger than that for case 1. We can confirm these SAR results corresponding to the field distributions in the previous section. The maximum allowable transmitting powers (MAP) for those cases are calculated with basic restrictions of the ICNIRP guidelines and are shown in Fig 7. This calculation suggests that the MAP of the HR-WPT system is 6.36 kW from the SAR10g result in the worst exposure scenario (case 2).

#### Various distances

**Fig 8:**
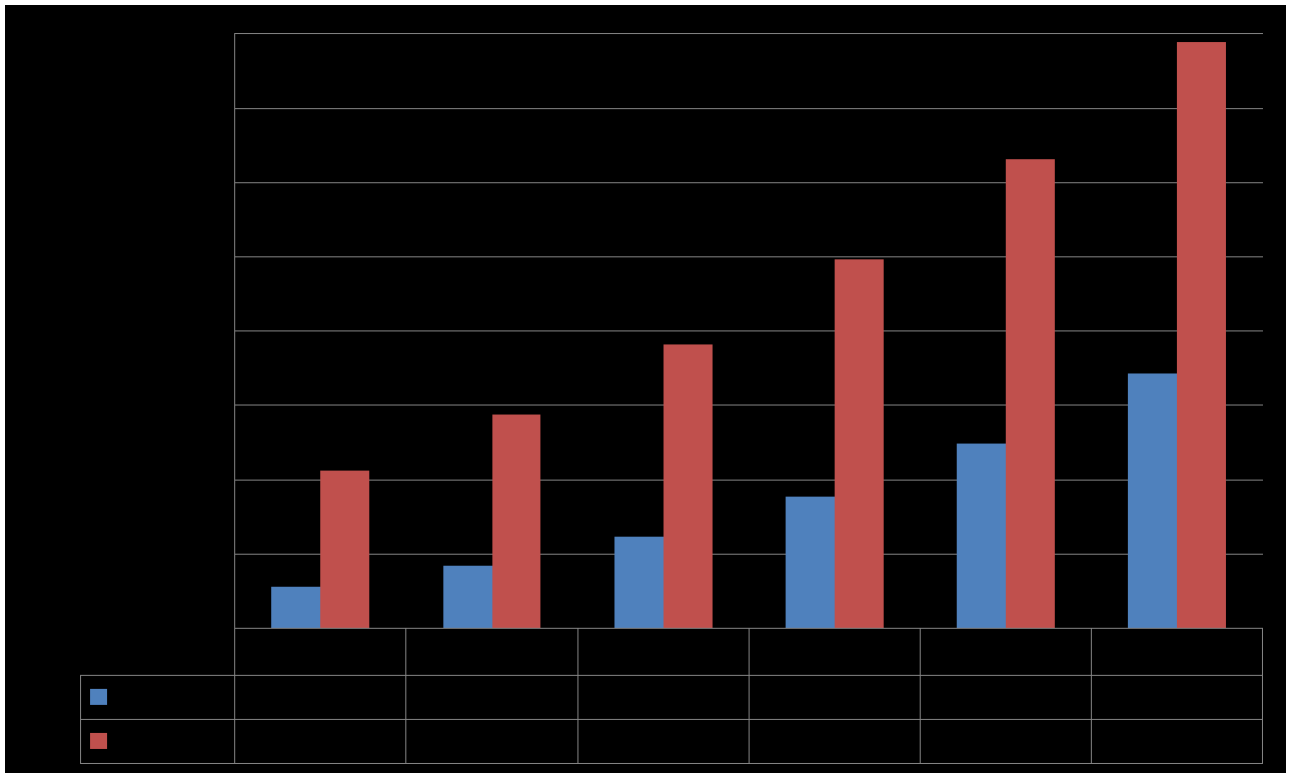
Maximum allowable power (MAP) along with distances between a human body and a highly resonant wireless power transfer system.

Dosimetry along with transition of distances (d) between the human body and the HR-WPT system for alignment condition is also conducted and compared to each other. The distances are 10 mm, 30 mm, 50 mm, 90 mm, and 110 mm. The SAR results are listed in Table 2. As we expect, the SARs decreases along with the distance. Fig 8 shows MAP along with distances between a human body and a HR-WPT system. At 10 mm distance of the worst exposure scenario in these cases, MAP is 27.8 kW, which is larger than that of misalignment case 2. The result represents that the factor of misalignment between the two transmitting and receiving coils is significant for electromagnetic field exposure.

**Table 2:** Specific absorption rate in a human body along with distances from a HR-WPT system.

Distance (mm)	SAR_peak_ (mW/kg)	SAR_10g_ (μW/kg)	SAR_wb_ (μW/kg)
10	3.14	360	3.77
Tissue name	skin	fat	-
30	2.07	238	2.77
Tissue name	skin	fat	-
50	1.37	162	2.09
Tissue name	skin	fat	-
70	0.93	113	1.61
Tissue name	skin	fat	-
90	0.66	80.3	1.27
Tissue name	skin	fat	-
110	0.47	58.3	1.01
Tissue name	skin	fat	-

SAR_peak_ represents the peak value of the SAR averaging volume of 2mm × 2mm × 2mm

SAR_10g_ represents the peak value of the SAR averaged over 10 g of tissue

SAR_wb_ represents the whole-body SAR

#### Coupling factors

Coupling factors *κ*_*peak*_ and κ are computed with calculated magnetic fields (*H*_*cal*_) in the center of a human body. From the coupling factors, the compensated magnetic fields, which mean maximum allowable external magnetic fields to satisfy reference levels, are derived. In this paper, the respect relaxation factors, ∝_*mp*_ and ∝_*m*_, are defined as inverse coupling factors 1/*κ*_*peak*_ and 1/κ. The results are listed in Table 3.

**Table 3:** Coupling factors and maximum allowable external magnetic fields.

Alignment & Misalignment	*κ*_*peak*_	κ	∝_*mp*_	∝_*m*_
Case 1	0.166	0.057	6.0	17.5
Case 2	0.218	0.074	4.6	13.4
Case 3	0.060	0.024	16.5	42.1
Case 4	0.051	0.020	19.5	50.3
Case 5	0.045	0.014	22.4	72.6
Distance (mm)				
10	0.234	0.079	4.3	12.6
30	0.197	0.067	5.1	15.0
50	0.166	0.057	6.0	17.5
70	0.142	0.049	7.0	20.2
90	0.124	0.043	8.1	23.1
110	0.109	0.038	9.2	26.1

As shown in Fig 3, near field from a HR-WPT system is non-uniform. Gradient of the field decreases along with distance from a HR-WPT system. Therefore, we can expect that coupling factor decreases along with distance as shown in Table 3. In case 2, which has the largest gradient of the field, coupling factor is the largest among cases at the same distance of 50 mm. The external magnetic field can be relaxed four to nigh times along with distance range of 10 mm to 110 mm, and four to twenty two times in the alignment and misalignment cases at 50 mm distance. Finally, in this calculation, the relaxation factor of the worst exposure scenario, four times, can be applied to the HR-WPT system for all cases.

## Conclusions

Dosimetry for a 13.56 MHz highly resonant wireless power transfer system (HR-WPT) has been conducted in the situations of misalignment and transition of distance between a human and the system. Anatomically realistic voxel human model was used to investigate specific absorption rates (SAR) in the model computed by the scattered field finite difference time domain method. From the results, maximum allowable transmitting powers and coupling factors in the various exposure scenarios are derived. It is found that the SAR for the misalignment case is larger than that for the alignment case. The results suggest that MAP is 6.36 kW and relaxation factor is 4 in this calculation.
